# Prognostic Models for 9-Month Mortality in Tuberculous Meningitis

**DOI:** 10.1093/cid/cix849

**Published:** 2017-09-26

**Authors:** Le Thi Phuong Thao, A Dorothee Heemskerk, Ronald B Geskus, Nguyen Thi Hoang Mai, Dang Thi Minh Ha, Tran Thi Hong Chau, Nguyen Hoan Phu, Nguyen Van Vinh Chau, Maxine Caws, Nguyen Huu Lan, Do Dang Anh Thu, Nguyen Thuy Thuong Thuong, Jeremy Day, Jeremy J Farrar, M Estee Torok, Nguyen Duc Bang, Guy E Thwaites, Marcel Wolbers

**Affiliations:** 1 Oxford University Clinical Research Unit, Ho Chi Minh City, Vietnam; 2 Nuffield Department of Medicine, University of Oxford, United Kingdom; 3 Hospital for Tropical Diseases; 4 Pham Ngoc Thach Hospital, Ho Chi Minh City, Vietnam; 5 Liverpool School of Tropical Medicine, Pembroke Place; 6 Department of Medicine, University of Cambridge, United Kingdom

**Keywords:** tuberculous meningitis, prognostic models, mortality, HIV

## Abstract

**Background:**

Tuberculous meningitis (TBM) is the most severe form of extrapulmonary tuberculosis. We developed and validated prognostic models for 9-month mortality in adults with TBM, with or without human immunodeficiency virus (HIV) infection.

**Methods:**

We included 1699 subjects from 4 randomized clinical trials and 1 prospective observational study conducted at 2 major referral hospitals in Southern Vietnam from 2001–2015. Modeling was based on multivariable Cox proportional hazards regression. The final prognostic models were validated internally and temporally and were displayed using nomograms and a Web-based app (https://thaole.shinyapps.io/tbmapp/).

**Results:**

951 HIV-uninfected and 748 HIV-infected subjects with TBM were included; 219 of 951 (23.0%) and 384 of 748 (51.3%) died during 9-month follow-up. Common predictors for increased mortality in both populations were higher Medical Research Council (MRC) disease severity grade and lower cerebrospinal fluid lymphocyte cell count. In HIV-uninfected subjects, older age, previous tuberculosis, not receiving adjunctive dexamethasone, and focal neurological signs were additional risk factors; in HIV-infected subjects, lower weight, lower peripheral blood CD4 cell count, and abnormal plasma sodium were additional risk factors. The areas under the receiver operating characteristic curves (AUCs) for the final prognostic models were 0.77 (HIV-uninfected population) and 0.78 (HIV-infected population), demonstrating better discrimination than the MRC grade (AUC, 0.66 and 0.70) or Glasgow Coma Scale score (AUC, 0.68 and 0.71) alone.

**Conclusions:**

The developed models showed good performance and could be used in clinical practice to assist physicians in identifying patients with TBM at high risk of death and with increased need of supportive care.

## Teaser

Prognostic models developed and validated using data from 1699 adults with tuberculous meningitis (TBM), with or without human immunodeficiency virus infection, performed well and could be used in clinical practice to identify patients with TBM at high risk of death.

Tuberculous meningitis (TBM) accounted for about 1%–5% of the 10.4 million new tuberculosis cases in 2015 and is the most severe manifestation of the disease, killing or disabling about half of those afflicted [1]. TBM is especially common in children and those infected with human immunodeficiency virus (HIV), in whom outcomes are poor [2]. In a trial conducted in Vietnam, approximately 30% of participants died during the 9-month study period, and 40% of the survivors had disabilities [3].

The British Medical Research Council (MRC) constructed the first TBM severity grades for use in the 1948 trial of streptomycin [4]. Patients were subdivided, based on clinical experience rather than statistical derivation, into “early” (no clinical signs of meningitis or focal neurology and fully conscious), “medium” (patient’s condition falling between early and advanced) and “advanced” (extremely ill, in deep coma). With the introduction of the Glasgow Coma Scale (GCS) in 1974, these categories were modified to the following grading system: grade I (GCS score 15; no focal neurological signs), grade II (GCS score 11–14 or 15 with focal neurological signs), or grade III (GCS score ≤10) [5]. This grading system has become the most widely used classification for TBM severity. Despite the age of the MRC scale, and its lack of statistical derivation, improved and robust prediction models for poor outcomes (death and/or neurological deficit) in TBM based on large cohort studies and rigorous statistical methods are still lacking. Among prognostic studies in TBM published in the last 20 years, the majority were based on a small number of subjects (ranging from 23 to 507), and modern prognostic modeling tools for handling missing data or model validation were rarely used [6–9].

The primary objective of this study was to develop and validate novel robust prognostic models for 9-month mortality in adult patients with TBM, with or without HIV coinfection. The models were based on a large data set of 1699 subjects (951 HIV uninfected, 748 HIV infected) enrolled in 4 randomized controlled trials and 1 prospective cohort study conducted in Vietnam. In addition, we compared the predictive performance of the developed models with the MRC grading system and the GCS, both widely used in the assessment of TBM severity.

## METHODS

### Study Population

#### Study Participants

The study population comprised subjects enrolled in 5 TBM studies conducted between 2001 and 2015 at 2 tertiary referral centers in Ho Chi Minh City, Vietnam: Pham Ngoc Thach Hospital and the Hospital for Tropical Diseases [3, 10–13]. Detailed descriptions of the studies have been published elsewhere [3, 10–13] and are summarized in Supplementary Table S1. All studies were approved by the Oxford Tropical Research, Hospital for Tropical Diseases, and Pham Ngoc Thach Hospital ethics committees.

#### Inclusion Criteria

The inclusion criteria for the 5 studies are provided in Supplementary Table S1. In brief, all 5 studies included adult subjects with a clinical diagnosis of TBM, defined as having >5 days of meningitis symptoms, nuchal rigidity, and cerebrospinal fluid (CSF) abnormalities suggestive of TBM; additional criteria were radiological evidence of tuberculosis on chest radiograph or brain scan or microbiological evidence of tuberculosis from specimens other than CSF. Diagnostic categories of definite, probable, or possible TBM were defined according to study-specific diagnostic criteria because the uniform TBM case definition became available only in 2010 [14]. All study participants were included in the pooled analysis except if they had a confirmed alternative diagnosis, a study drug administration error, or an unknown HIV status.

#### Laboratory Investigation

CSF specimens were stained and cultured by standard methods for pyogenic bacteria, mycobacteria, and fungi. In the last study [3], CSF was also tested with the Xpert MTB/RIF assay (Cepheid). Isolates of *M. tuberculosis* were tested for susceptibility to isoniazid, rifampin, ethambutol, and streptomycin with the mycobacterial growth indicator tube method [15]. Baseline peripheral blood CD4 cell counts were measured for all HIV-infected adults, using flow cytometry.

#### Antituberculosis and Adjunctive Treatment

Unless study participants were randomized to an experimental antituberculosis treatment, participants received a standard antituberculosis regimen consisting of isoniazid (5 mg/kg/d; maximum, 300 mg/d), rifampin (10 mg/kg/d; maximum, 600 mg/d), pyrazinamide (25 mg/kg/d; maximum, 2 g/d) and ethambutol (20 mg/kg/d; maximum, 1.2 g/d) or streptomycin (20 mg/kg/d; maximum, 1 g/d) for 3 months, followed by rifampin and isoniazid at the same doses for another 6 months. Since 2005, all patients received adjunctive dexamethasone for the first 6–8 weeks of treatment [3].

### Primary Outcome

The primary end point was overall survival during a 9-month follow-up period. Patients without documented death during the follow-up period were censored at 9 months or at the last date they were known to be alive, whichever was earlier.

### Candidate Predictors

Candidate predictors were initially selected based on clinical judgment, their status as established risk factors in previous publications [6–9, 16–18], and completeness of the data. The number of predictors was further restricted based on a rule of thumb of requiring at least 10 events for each included predictor variable or degree of freedom [19]. The final list of candidate predictors is presented in Supplementary Table S2. For all laboratory parameters and radiology assessments, we used the value recorded closest to enrollment (up to ±7 days from enrollment). Of note, CSF total white blood cell count and CSF total lymphocyte count were strongly correlated; therefore, only CSF lymphocyte count was included as candidate predictor. 

Resistance is known to be an important independent predictor of death in subjects with TBM [20, 21]. However, unless earlier isolates are available or rapid molecular tests are performed, information about a patients’ tuberculosis drug susceptibility is not available at enrollment. The main models therefore did not include resistance, but models with resistance were depicted in the Supplementary Materials. The cohort variable was included in the full model to represent the change in patient management, treatment, and standard of health care over the 15-years time span of the included studies. We decided to construct separate prognostic models for the HIV-uninfected and HIV-infected TBM populations, because they are clinically distinct populations and we expected that predictors may differ between them.

### Statistical Analysis

Full details of the statistical analysis are described in the Supplementary Appendix S1. In brief, incomplete data were multiply imputed using multivariable imputation by chained equations (MICE) [22]. The statistical model of choice was multivariable Cox proportional hazards regression including all prespecified candidate predictors. We tested for potential nonlinear effects of continuous candidate predictors and for interactions, that is, effect modifications, between age and other candidate predictors. Two variable selection methods were used to simplify the model: (1) backward stepwise selection with a stopping rule based on Akaike’s information criterion and (2) the least absolute shrinkage and selection operator (LASSO) [23]. 

We combined model selection with bootstrapping and based the final models on the most frequently (≥60%) included variables across all imputed and bootstrap data sets [24]. We used the area under the cumulative/dynamic receiver operating characteristic (ROC) curve (AUC) [25] for 9-month mortality risk prediction to assess the discrimination of the models and calibration plots [26] and to visually assess how closely the predicted mortality probabilities agree with the observed mortality probabilities. Internal bootstrap validation was performed to correct measures of model performance for overfitting. In addition, we performed temporal validation, wherein data from the most recent trial served as the test data set to validate models developed based on earlier studies. The final prognostic models, that is, the variable-selected models with the highest AUC, were implemented in a Web-based mortality calculator and graphically depicted using nomograms [26]. All analyses were conducted using R software, version 3.3.1 [27].

## RESULTS

### Baseline Characteristics of Study Participants

The 5 studies included a total of 1734 subjects, of which 1699 were included for prognostic modeling (Supplementary Figure S1). The reasons for excluding 35 subjects were a confirmed other diagnosis (n = 16), unknown HIV status (n = 16), or a study drug administration error (n = 3).

Table 1 presents the baseline characteristics of all included subjects; 951 were HIV uninfected (56.0%), and 748 (44.0%) were HIV infected. The median age was 34 years (interquartile range, 27–45 years), and 823 subjects (48.4%) had definite, microbiologically confirmed TBM. The MRC grade was I in 588 subjects (34.6%), II in 743 (43.8%), and III in 367 (21.6%). Among HIV-infected subjects, the median CD4 cell count was 41 cells/μL, and 124 (16.6%) were receiving antiretroviral therapy (ART) at baseline. Compared with HIV-uninfected subjects, those infected with HIV tended to be younger (median age, 31 vs 40 years), were more frequently male (85.6% vs 62.3% male) and were more likely to be MRC grade III (25.6% vs 18.5%).

**Table 1.** Baseline Characteristics of Patients With Tuberculous Meningitis Included in the Pooled Database, Overall and By Human Immunodeficiency Virus Status

Characteristic	All Patients (N = 1699)		HIV Uninfected (n = 951)		HIV Infected (n = 748)	
	Total No.^a^	No. (%)^b^	Total No.^a^	No. (%)^b^	Total No.^a^	No. (%)^b^
Cohort	1699		951		748	
Dexamethasone trial		534 (31.4)		436 (45.9)		98 (13.1)
Fluoroquinolone trial		56 (3.3)		53 (5.6)		3 (0.4)
TBM HIV cohort		58 (3.4)		0 (0)		58 (7.7)
ART timing trial		248 (14.6)		0 (0)		248 (33.2)
Intensified treatment trial		803 (47.3)		462 (48.6)		341 (45.6)
Age, median (IQR), y	1698	34 (27–45)	951	40 (27–56)	747	31 (26–35)
Female sex	1699	514 (30.25)	951	406 (42.7)	748	108 (14.4)
Weight, median (IQR), kg	1695	46 (41–51)	951	46 (42–52)	744	45 (40–50)
Received dexamethasone treatment	1699	1436 (84.5)	951	742 (78.0)	748	694 (92.8 )
Receiving ART at enrollment	745	124 (16.6)	…	…	745	124 (16.6)
MRC grade^c^	1698		951		747	
Grade I		588 (34.6)		327 (34.4)		261 (34.9)
Grade II		743 (43.8)		448 (47.1)		295 (39.5)
Grade III		367 (21.6)		176 (18.5)		191 (25.6)
Illness duration at study entry, median (IQR), d	1685	15 (10–30)	948	15 (10–26)	737	15 (9–30)
History of previous tuberculosis treatment	1658	236 (14.2)	922	88 (9.5)	736	148 (20.1)
Focal neurological signs present	1683	818 (48.6)	951	517 (54.4)	732	301 (41.1)
Temperature, median (IQR), °C	1697	37.6 (37.2–38.5)	950	37.7 (37.2–38.5)	747	37.5 (37.2–38.5)
Occurrence of seizures	1685	50 (3.0)	950	25 (2.6)	735	25 (3.4)
Laboratory values, median (IQR)						
Plasma sodium, mmol/L	1537	129 (124–133)	843	130 (125–134)	694	127 (123–132)
CSF lymphocyte count, cells/μL	1614	85 (27.6–197.5)	919	94 (32.5–200)	695	73 (23.3–188.8)
CSF protein, g/L	1624	1.3 (0.70–1.94)	909	1.2 (0.7–2.0)	715	1.3 (0.7–1.9)
CSF glucose, mmol/L	1636	1.60 (1.05–2.30)	920	1.52 (1.00–2.30)	716	1.70 (1.17–2.33)
Ratio of CSF to blood glucose	1470	0.30 (0.21–0.41)	830	0.30 (0.20–0.40)	640	0.30 (0.21–0.42)
Microbiologically confirmed/definite TBM	1699	823 (48.4)	951	361 (38.0)	748	462 (61.8)
Resistance^d^	1699		951		748	
No isoniazid or rifampin resistance		479 (28.2)		203 (21.4)		276 (36.9)
Isoniazid monoresistance		172 (10.1)		63 (6.6)		109 (14.6)
Rifampin monoresistance/MDR		35 (2.1)		10 (1.1)		25 (3.3)
Unknown resistance		1013 (59.6)		675 (71.0)		338 (45.2)
Miliary tuberculosis present on chest radiograph	1525	279 (18.3)	890	165 (18.5)	635	114 (17.9)
Peripheral blood CD4 count, median (IQR), cells/μL	646	41 (16–108)	…	…	646	41 (16–108)

Abbreviations: ART, antiretroviral therapy; CSF, cerebrospinal fluid; HIV, human immunodeficiency virus; IQR, interquartile range; MDR, multidrug resistance; MRC, Medical Research Council; TBM, tuberculous meningitis.

^a^No. of subjects with nonmissing data for the respective characteristic.

^b^Data represent no. (%) of subjects unless otherwise specified.

^c^MRC grade I is defined as Glasgow Coma Scale (GCS) score 15 with no focal neurological signs; grade II, GCS score 11–14, or 15 with focal neurological signs; grade III, GCS score ≤10.

^d^Isoniazid monoresistance is defined as resistance to isoniazid but not to rifampin; MDR, as resistance to at least isoniazid and rifampin; unknown resistance, as drug-susceptibility test results not available. In all categories, resistance to other drugs may be present.

**Figure 1.**
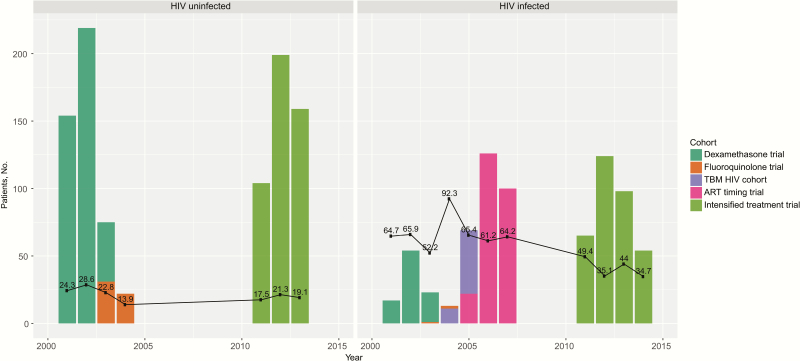
Annual recruitment into the 5 contributing studies by human immunodeficiency virus status. Black lines show estimated 9-month mortality rates (as percentages) for each calendar year, based on the Kaplan-Meier method. Abbreviations: ART, antiretroviral therapy; HIV, human immunodeficiency virus; TBM, tuberculous meningitis.

During 9-month follow-up, 219 of 951 HIV-uninfected subjects with TBM (23.0%) died, compared with 384 of 748 HIV-infected subjects (51.3%). Among the 1096 survivors, 998 (91.1%) were followed up for ≥260 days. Yearly recruitment into the 5 studies and observed mortality rate by HIV status are shown in Figure 1. The figure displays a decline in mortality rate over time, which is particularly pronounced in HIV-infected subjects. In this subpopulation, the estimated 9-month mortality rate dropped from 52%–92% during 2001–2007 to 35%–50% during 2011–2015. Coincidently, the number of study participant receiving ART at enrollment was only 7 of 407 (1.7%) in the first period and increased to 117 of 341 (34.3%) in the second period.

### Prognostic Models

Univariable analyses of the effect of all candidate predictors for survival are shown in Supplementary Tables S3 and S4. Results of the multivariable analyses are shown in Table 2 and Supplementary Table S5. The final models identified higher MRC disease severity grade and lower CSF lymphocyte cell counts as risk factors for death in both HIV-uninfected and HIV-infected subjects (Table 2). Importantly, the mortality rate in MRC grade III (GCS score ≤10) was 3-fold higher in HIV-uninfected patients and 4-fold higher in HIV-infected patients compared with MRC grade I (GCS score 15; no focal neurological signs). Mortality rates for MRC grade II (GCS score 11–14, or 15 with focal neurological signs) was intermediate in both populations.

**Table 2.** Final Cox Regression Models for 9-Month Survival in Each Human Immunodeficiency Virus Population^a^

Variable	HIV-Uninfected TBM Population						HIV-Infected TBM Population					
	Full Model			Final Model^b^			Full Model			Final Model^b^		
	HR	95% CI	*P* Value	HR	95% CI	*P* Value	HR	95% CI	*P* Value	HR	95% CI	*P* Value
Age (per +10 y)	1.24	1.15–1.34	<.001	1.24	1.15–1.34	<.001	0.99	.85–1.16	.89	…	…	…
Male sex	1.07	.79–1.44	.67	…	…	…	0.95	.70–1.30	.77	…	…	…
Weight (per +10 kg)	0.88	.73–1.06	.17	…	…	…	0.72	.61–.85	<.001	0.73	.63–.85	<.001
MRC grade^c^												
Grade I	1	…	…	1	…	…	1	…	…	1	…	…
Grade II	1.36	.87–2.13	.17	1.41	.9–2.19	.13	1.71	1.24–2.35	.001	1.88	1.43–2.48	<.001
Grade III	2.97	1.83–4.83	<.001	3.05	1.92–4.86	<.001	3.76	2.69–5.26	<.001	4.08	3.07–5.41	<.001
Illness duration at study entry (d)^d^	1.03	.91–1.17	.61	…	…	…	1.02	.93–1.11	.73	…	…	…
History of previous tuberculosis treatment	1.46	1.00–2.13	.05	1.57	1.09–2.26	.02	1.26	.97–1.65	.09	…	…	…
Focal neurological signs present	1.80	1.22–2.64	.003	1.65	1.15–2.39	.007	1.15	.87–1.53	.33	…	…	…
Temperature (°C)	0.96	.79–1.17	.71	…	…	…	1.09	.96–1.24	.18	…	…	…
Occurrence of seizures	0.96	.41–2.22	.92				0.95	.58–1.56	.83			
No dexamethasone: treatment	1.67	1.15–2.40	.006	1.97	1.46–2.67	<.001	1.12	.68–1.85	.65	…	…	…
Plasma sodium (per +10 mmol/L)^e^	1.01	.85–1.21	.89	…	…	…	…	…	<.001	…	…	<.001
Plasma sodium, 135 vs 125 mmol/L	1.01	.85–1.21	…	…	…	…	1.09	.91–1.31	…	1.07	.90–1.28	…
Plasma sodium, 115 vs 125 mmol/L	0.99	.83–1.17	…	…	…	…	1.63	1.31–2.04	…	1.06	1.29–2.00	…
CSF lymphocyte count (cells/μL)^d^	0.88	.82–.94	<.001	0.86	.81–.92	<.001	0.93	.87–.99	.02	0.93	.88–.98	.004
CSF protein (g/L)^d^	0.95	.84–1.07	.38	…	…	…	0.95	.85–1.06	.37	…	…	…
CSF glucose (mmol/L)	1.03	.87–1.23	.70	…	…	…	1.08	.98–1.19	.10	…	…	…
Ratio of CSF to blood glucose^d^	1.02	.81–1.28	.88	…	…	…	0.91	.77–1.08	.30	…	…	…
Miliary tuberculosis present on chest radiograph	0.82	.56–1.18	.29	…	…	…	0.95	.70–1.29	.73	…	…	…
Peripheral blood CD4 (cells/μL)^d^	…	…	…	…	…	…	0.91	.85–.98	.01	0.9	.84–.96	.002
Receiving ART at enrollment	…	…	…	…	…	…	0.87	.60–1.26	.45	…	…	…
Cohort												
Intensified trial	1	…	…	…	…	…	1	…	…	1	…	…
Dexamethasone trial	1.46	.94–2.27	.09	…	…	…	1.96	1.23–3.14	.005	1.72	1.25–2.37	<.001
Fluoroquinolone trial	1.12	.51–2.46	.77	…	…	…	…	…	…	…	…	…
TBM HIV cohort	…	…	…	…	…	…	2.58	1.63–4.06	<.001	2.64	1.81–3.84	<.001
ART timing trial	…	…	…	…	…	…	1.78	1.28–2.47	<.001	1.71	1.34–2.19	<.001

Abbreviations: ART, antiretroviral therapy; CI, confidence interval; CSF, cerebrospinal fluid; HIV, human immunodeficiency virus; HR, hazard ratio. MRC, Medical Research Council; TBM, tuberculous meningitis.

^a^Estimates were pooled across multiply imputed data sets.

^b^The final model for the HIV-uninfected population was selected using the least absolute shrinkage and selection operator (LASSO) method. The final model for the HIV-infected population was selected using stepwise backward model selection. The 95% CIs and *P* values for final models do not take into account the uncertainty of model selection.

^c^MRC grade I is defined as Glasgow Coma Scale (GCS) score 15 with no focal neurological signs); grade II, GCS score 11–14, or 15 with focal neurological signs); grade III, GCS score ≤10.

^d^HR per 2-fold increase.

^e^In HIV-infected subjects, the effect of sodium levels on mortality rates was significantly nonlinear and modeled with a restricted cubic spline function with 2 *df*. To simplify interpretation of the corresponding regression coefficients, only HRs for 2 derived sodium contrasts from that model are given. *P* values for sodium values In the HIV-infected population are based on overall Wald tests of the restricted cubic spline function.

In HIV-uninfected subjects, older age, previous tuberculosis, not receiving adjunctive dexamethasone, and focal neurological signs were additional predictors of higher mortality risk. Of note, the mortality rate was increased by >50% in patients with previous tuberculosis and those with focal neurological signs, and almost doubled for patients who did not receive dexamethasone.

In the HIV-infected population, lower weight, lower peripheral blood CD4 cell count, and abnormal plasma sodium were additional predictors of higher mortality. Plasma sodium had a significant nonlinear association with survival, and both decreased and elevated sodium values were associated with a higher predicted mortality rate compared with intermediate value values. Ongoing ART at enrollment was associated with a more favorable outcome in univariable analysis (Supplementary Table S4) but was no longer significantly associated with survival after adjustment for other factors. There was no clear evidence that age modified the effect of any of the other predictors (overall interaction test, *P* = .14 in HIV-uninfected and *P* =.10 in HIV-infected subjects).

### Model Validation

The final models showed good discrimination between TBM survivors and deaths at 9 months; the corresponding AUCs in internal validation (corrected for overfitting) were 0.77 in the HIV-uninfected and 0.78 in the HIV-infected population (Table 3). The addition of drug resistance as a covariate resulted in only a slightly improved model discrimination, with AUC values ranging from 0.77 to 0.79 (Supplementary Table S7).

**Table 3.** Discrimination of Candidate Models for Tuberculous Meningitis Mortality By Human Immunodeficiency Virus Population Measured By Area Under the Curve at 9 Months

Model	Internal Validation		Temporal Validation: AUC (95% CI)
	Apparent AUC (95% CI)^a^	Optimism-Corrected AUC^b^	
HIV-uninfected population			
Full model	0.79 (.76–.83)	0.76	0.77 (.72–.83)
Model selected by stepwise backward model selection	0.78 (.74–.81)	0.76	0.77 (.71–.82)
Model selected by LASSO method^c^	0.78 (.75–.82)	0.77	0.82 (.77–.87)
MRC grade^d^	0.66 (.62–.70)	0.66	0.70 (.64–.75)
GCS	0.68 (.64–.72)	0.68	0.68 (.62–.75)
HIV-infected population			
Full model	0.79 (.75–.83)	0.77	0.76 (.70–.82)
Model selected by stepwise backward model selection^c^	0.79 (.75–.82)	0.78	0.73 (.67–.79)
Model selected by LASSO method	0.78 (.74–.82)	0.77	0.75 (.69–.81)
MRC grade^d^	0.70 (.66–.74)	0.70	0.69 (.63–.75)
GCS	0.71 (.67–.75)	0.71	0.68 (.62–.74)

Abbreviations: AUC, area under the cumulative/dynamic receiver operating characteristic curve; CI, confidence interval; GCS, Glasgow Coma Scale; HIV, human immunodeficiency virus; LASSO, least absolute shrinkage and selection operator; MRC, Medical Research Council.

^**^a^**^Performance estimated directly from the original 45 imputed data sets used to develop the prediction models.

^b^Adjusted performance corrected for overoptimism through internal bootstrap validation.

^c^Final simplified model.

^d^MRC grade I is defined as GCS score 15 with no focal neurological signs; grade II, GCS score 11–14, or 15 with focal neurological signs; and grade III, GCS score ≤10.

In internal validation, the models showed good agreement between predicted and observed mortality (Supplementary Figure S2*A* and S2*C*). In temporal validation, calibration in the HIV-uninfected groups remained satisfactory, whereas in the HIV infected group models developed based on earlier studies systematically overestimated the actual mortality in the most recent trial (Supplementary Figure S2*B* and S2*D*).

**Figure 2.**
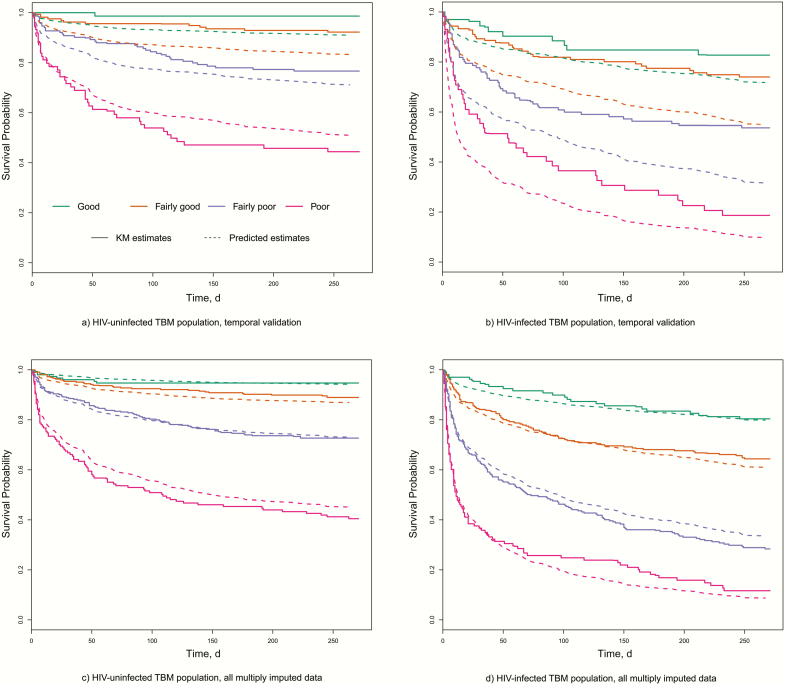
Comparison between predicted survival with the final models and observed Kaplan-Meier estimates. Risk groups are defined using cutoff points at the 16th, 50th, and 84th percentiles of the prognostic index as generated by the final models (defining “good,” “fairly good,” “fairly poor,” and “poor” prognostic subgroups). (*A*) HIV-uninfected TBM population, temporal validation; (*B*) HIV-infected TBM population, temporal validation; (*C*) HIV-uninfected TBM population, all multiply imputed data; (*D*) HIV-infected TBM population, all multiply imputed data. Abbreviations: HIV, human immunodeficiency virus; KM, Kaplan-Meier; TBM, tuberculous meningitis.

Figure 2 shows predicted survival curves of the final simplified models in 4 risk groups defined using cutoff points at the 16th, 50th, 84th percentiles of the prognostic index generated by these models [28]. They show strong prognostic separation across risk strata, good agreement between predicted survival curves and Kaplan-Meier estimates in the full data set, and mild to substantial risk overestimation in temporal validation in HIV-uninfected and HIV-infected subjects

### Comparison of Prognostic Models With MRC Grade and GCS Alone

**Figure 3.**
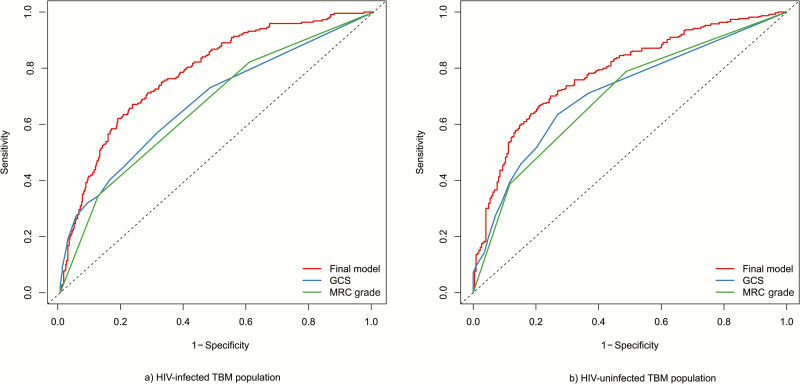
Cumulative/dynamic receiver operating characteristic curves (apparent estimate) for mortality rates evaluated at 9 months for the final prognostic model, Glasgow Coma Scale (GCS) score, and Medical Research Council (MRC) grade in the tuberculous meningitis populations with or without human immunodeficiency virus infection. MRC grade I is defined as GCS score 15 with no focal neurological signs; grade II, GCS score 11*–*14, or 15 with focal neurological signs; grade III, GCS score ≤0. (*A*) HIV-uninfected TBM population; (*B*) HIV-infected TBM population. Abbreviations: GCS, Glasgow Coma Scale; HIV, human immunodeficiency virus; MRC, Medical Research Council; TBM, tuberculous meningitis.

MRC grade and GCS predicted mortality rate similarly well (AUC, 0.66–0.71) (Table 3). The corresponding ROC curves for MRC grade, GCS score, and the final models are shown in Figure 3. MRC grade and GCS score were both clearly inferior to the developed multivariable models in terms of discrimination (for all comparisons, *P* < .001 for tests of equality between AUC values), whereas discrimination did not differ significantly between MRC grade and GCS score (*P* = .23 in HIV-uninfected and *P* = .19 in HIV-infected subjects).

### Nomograms and Web App for Mortality Rate Prediction

**Figure 4.**
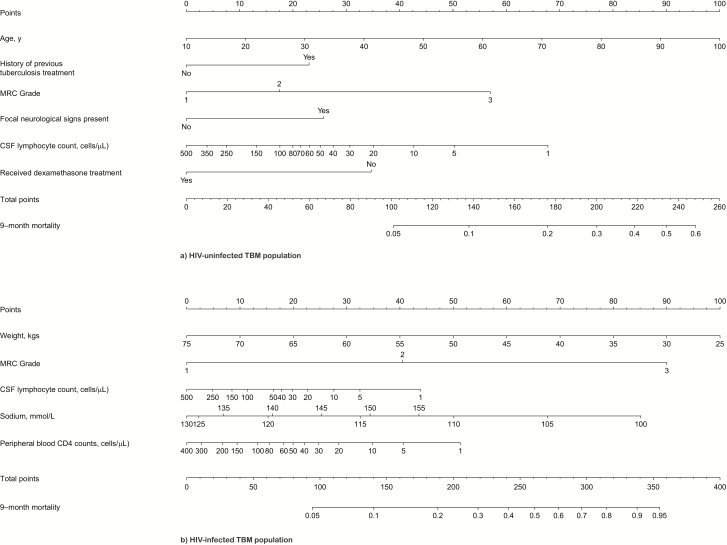
Nomograms for the prediction of 9-month mortality based on the final prognostic models for the tuberculous meningitis populations without (*A*) or with (*B*) human immunodeficiency virus (HIV) infection. To derive a prediction, locate the value of each predictor on the corresponding variable line, read the corresponding points assigned on the 0*–*100 scale, and sum all of these points to a total point score. Then read the result on the “total points” scale and its corresponding prediction below. For HIV-infected population, the cohort variable was chosen as the most recent trial [3], because this is most relevant for future prediction. Abbreviations: CSF, cerebrospinal fluid; HIV, human immunodeficiency virus; MRC, Medical Research Council; TBM, tuberculous meningitis.

Figure 4 visualizes the final models as nomograms. For clinical use, this model has also been implemented as a user-friendly Web app (available at https://thaole.shinyapps.io/tbmapp/). We also generated nomograms for use when resistance information is available (Supplementary Figure S3). Of note, for creation of the nomogram and the Web app in the HIV-infected population, the cohort variable was chosen as our most recent trial [3], because this is most relevant for future prediction.

## DISCUSSION

We developed and validated prognostic models for 9-month mortality after TBM diagnosis in a large data set of 951 HIV-uninfected and 748 HIV-infected patients with TBM. We found that a higher MRC grade and lower CSF lymphocyte cell counts. Predictors of death in both groups. MRC grade assesses the severity of neurological impairment and numerous studies have shown its strong association with poor outcome [29, 30]. The predictive value of lower CSF lymphocyte counts is intriguing. Others have recently found that higher CSF neutrophil count predicted death from TBM [31]; both findings reflect that the importance of neuroinflammation in determining outcome from TBM is poorly understood.

Additional risk factors for mortality in HIV-uninfected patients were older age, previous tuberculosis treatment, not receiving dexamethasone, and having focal neurological signs. In HIV-infected subjects, additional risk factors were lower weight, lower CD4 cell count, and abnormal plasma sodium level. These finding are consistent with results from previous published studied conducted in India [32], Hong Kong [17], Taiwan [8], Turkey [33], South Africa [34], and Brazil [35]. The inclusion of different predictors in the 2 HIV populations may suggest important differences in disease pathogenesis and outcomes between these subgroups.

The main analysis did not include drug resistance as a covariate because this information is often not available at enrollment. Alternative models including drug resistance showed only relatively little improvement in model performance, especially in HIV-uninfected subjects. However, we note that the low prevalence in our cohort of multidrug-resistant tuberculosis, an important predictor of poor outcome in TBM [20, 21], may have contributed to this finding.

The final prediction models were carefully developed and validated with proper statistical methods [36]. They showed good discrimination between TBM survivors and patients who died in both internal and temporal validation, with substantial improvement over the MRC grade or the GCS score alone. Although our models had excellent calibration in internal validation, they overestimated mortality rates in temporal validation where the model was developed from earlier studies and tested on subjects included in the most recent trial. This overestimation was mild in HIV-uninfected subjects but substantial in HIV-infected subjects. It may be explained by improvements in treatment, especially the availability of ART, and patients’ supportive care over time. Importantly, because ART did not become widely available in Vietnam until 2005 [37] and receiving ART was an exclusion criterion for the ART timing trial, the intensified treatment trial [3] was the only study that included subjects receiving ART at TBM diagnosis. Therefore, the effect of ART was neglected in the temporal validation, which may have contributed to the observed overestimation. Temporal heterogeneity was addressed by including the cohort as a covariate in the statistical regression models. Survival for future patients is anticipated to be close to predictions adjusted to our most recent study.

Our study has several limitations, mostly owing to the characteristics of our database. First, CSF lactate, which may be an important risk factor [38], was not taken into account in our final model because it was missing in >40% of subjects. Second, previous studies suggested that leukotriene A4 hydrolase (LTA4H) genotype is a determinant of the inflammatory response in HIV-uninfected adults with TBM and consequently might predict who benefits from adjunctive corticosteroid treatment [39]. This factor may therefore influence survival in HIV-uninfected subjects with TBM. Because the information of LTA4H genotype was not available for all included studies, we did not consider this covariate in our analyses. Third, the models were developed and validated based on data from 2 large hospitals in Southern Vietnam only. Thus, it would be desirable to validate and possibly improve our models in an independent external database. Finally, our models included only risk factors available at TBM diagnosis. However, changes in biomarkers repeatedly measured during the disease course may carry important information that could allow an initial prognosis to be updated and improved during tuberculosis treatment. The value of such longitudinally measured biomarkers will be examined in a future research project.

Our final models were displayed as nomograms and implemented in a user-friendly Web-based risk calculator (https://thaole.shinyapps.io/tbmapp/) for use in clinical practice to improve prognostic stratification in patients with TBM. Patients at high risk could be identified early and then monitored more strictly to receive appropriate counseling or supportive care, or enrolled in future clinical trials exploring novel treatments targeted to severe TBM.

Our models are based on one of the largest sources of prospectively collected clinical data for TBM disease worldwide. The final prognostic models included variables that are usually available for patients with TBM. Overall, our prediction models have good discrimination and calibration performance, and they clearly outperformed the MRC grading score.

## Supplementary Data

Supplementary materials are available at *Clinical Infectious Diseases* online. Consisting of data provided by the authors to benefit the reader, the posted materials are not copyedited and are the sole responsibility of the authors, so questions or comments should be addressed to the corresponding author.

## Supplementary Material

Supplemental Data

## Notes

***Acknowledgments.*** We thank all the study physicians and nurses of Pham Ngoc Thach Hospital and the Hospital for Tropical Diseases for their attentive care to the patients, and the staff of the Clinical Trials Unit and Tuberculosis Group of the Oxford University Clinical Research Unit for their enduring support; we also gratefully acknowledge all the patients and their relatives for their participation in the included studies.

***Financial support.*** This work was supported by the Academy of Medical Sciences and the Health Foundation (Clinician Scientist Fellowship to M. E. T.), the National Institute of Health Research Cambridge Biomedical Research Centre (M. E. T), and a Wellcome Trust Intermediate Fellowship (grant number WT097147MA) to J.D.

***Potential conflicts of interest.*** All authors: No reported conflicts of interest. All authors have submitted the ICMJE Form for Disclosure of Potential Conflicts of Interest. Conflicts that the editors consider relevant to the content of the manuscript have been disclosed.
