# Supplementary material for: Prognostic Models for 9-Month Mortality in Tuberculous Meningitis
Source: Clin Infect Dis. 2017 Sep 26;66(4):523–32. doi: 10.1093/cid/cix849 (PMC5850565; doi:10.1093/cid/cix849)

# Supplementary appendix S1: Additional details regarding statistical methods

### Transformation of covariates

The duration of illness at enrolment, CSF lymphocyte count, CSF protein, ratio of glucose level in blood and glucose level in CSF, and peripheral blood CD4 cell count had a pronounced right-skewed distribution and were log-2 transformed prior to the analysis. We added 1 unit to the CD4 cell count before applying the transformation to avoid taking the log of zero.

### Treatment of missing data

Incomplete data in the pooled database were addressed by multiple imputation. Assuming that data are missing at random (MAR), we employed multivariable imputation by chained equations (mice) to generate imputed data sets [1] . Predictive mean matching was used to impute missing continuous variables, and logistic regression was employed to impute missing binary variables. The imputation models comprised all predictor variables as well as the survival outcomes. The latter was included as a combination of event status and the standard Nelson-Aalen estimate of the cumulative hazard function [2]. Other relevant variables such as hospital and glucose level in blood were also included in the imputation models. Forty-five imputed data sets (denoted as $Imp_{i}, i=1,\ldots,45$) were generated for the analysis, based on the percentage of incomplete cases[3]. Estimates from multiply imputed data sets were combined using Rubin’s rule[4]. All subsequent analyses were performed the multiply imputed data sets, except for the univariable analysis which was based solely on the complete cases for each predictor.

### Model development, validation, and assessment

*Statistical models for prediction*: The statistical model of choice was a multivariable Cox proportional hazards regression. We first fitted a full model with all pre-specified prognostic variables with a linear term for all continuous variables and no interactions. Next, we tested for non-linear relationships of the continuous variables by modeling them as restricted cubic spline functions with two degrees of freedom or, equivalently, three knots [5]. Knots were placed at the 10^th^, 50^th^, and 90^th^ percentile distribution of each variable. If the spline model was significantly superior (p value ≤0.05) to a model with a linear term only based on a Wald-test, we included the significant non-linear terms. Subsequently, to assess whether age modified the effect of other predictor variables, we tested for the presence of an interaction between age and all other covariates jointly using an overall Wald test. Again, if the overall test was significant, we included the significant interactions in the full model.

Next, two variable selection methods were used: i) stepwise backwards model selection with a stopping rule based on Akaike's information criterion, and ii) the Least Absolute Shrinkage and Selection Operator (lasso), in which the optimal penalty was determined based on 10-fold cross validation with the partial likelihood as the criterion function [6]. We chose the largest penalty that was within one standard error from the optimal one. Both methods were applied to 200 bootstrap samples, which were randomly drawn with replacement from each imputed dataset. The proportion of times that each variable appeared in 45 (imputations)*200 (bootstrap samples) = 9000 models was calculated and a variable was selected if this proportion was >60% [7]. For the lasso method, a variable represented by more than two parameters (e.g. a factor with >2 levels) was retained as a whole if one of its components was selected. The final models were then obtained by refitting the selected predictors to each of the 45 imputed datasets ($Imp_{i}, i=1,\ldots,45$)and combining results using Rubin's rule [4,8].

*Model performance*: We used the area under the cumulative/dynamic ROC curve (AUC)[9] at 9 months to assess the discrimination performance of the models, and a calibration plot [10] to visually assess how closely the predicted survival probabilities agreed with the observed probabilities.

*Model validation*: We performed both internal and temporal validation.
In internal validation, we bias-corrected performance measures by subtracting the estimated ‘optimism’, i.e. the difference between the performance of the model on the training data which was used to derive the model (apparent performance), and the performance of the model on a test/validation sample (test performance). The ‘optimism’ was calculated using the bootstrap by designating the original sample as the validation sample and bootstrap samples as training data sets as proposed by [10] (section 5.3.5, page 114). Specifically, to account for multiply imputed data, the method was implemented as follows: We first drew 100 bootstrap samples with replacement from the original dataset. For each bootstrap sample, we repeated the imputation (denoted as $Imp_{i}^{*} , i=1,\ldots,45$) and variable selection steps as described above. The obtained final models from each bootstrap sample were validated on the 45 bootstrap imputed data sets ($Imp_{i}^{*}, i=1,\ldots,45$) which were used to derive the prediction model to quantify the apparent performance, and on the 45 original imputed datasets ($Imp_{i} , i=1,\ldots,45$) to quantify the test performance. The difference between the apparent and test performance for each candidate model was then averaged across bootstrap samples and imputed datasets to get an estimate of the overall optimism which was used to bias-correct apparent performance measures for over-fitting[10].
In temporal validation, data from the most recent study, the intensified treatment trial, served as the test dataset to validate models that were developed based on data taken from the four earlier studies. Here, we performed the imputation step on the test and the validation dataset independently. Since none of the HIV-infected patients received ART at enrollment in studies other than 05TB, this covariate was not included in the temporal validation. The cohort variable was also excluded from the temporal validation.

*Model presentation and statistical software*: The final prognostic models were graphically depicted by nomograms [10]. All analyses were conducted using R version 3.3.1 [11] and the following packages: **survival** [12], **rms** [13], **mice** [14], **glmnet** [15], **mgcv** [16], **pec** [17], and **timeROC** [18]. The web app for risk prediction was programmed using **shiny** [19]

# References

1. van Buuren S, Groothuis-Oudshoorn K. mice: Multivariate Imputation by Chained Equations in R. J. Stat. Softw. **2011**; 45:1–67.

2. White IR, Royston P. Imputing missing covariate values for the {C}ox model. Stat Med **2009**; 28:1982–1998.

3. Harrell F, Lee KL, Mark DB. Tutorial in biostatistics multivariable prognostic models : issues in developing models , evaluating assumptions and adequacy , and measuring and reducing errors. **1996**; 15:361–387.

4. Little RJA, Rubin DB. Statistical Analysis with Missing Data. Wiley-Blackwell, 2002.

5. Terry M. Therneau PMG. Modeling Survival Data: Extending the {C}ox Model. Springer New York, 2013.

6. Hastie T, Tibshirani R, Friedman J. The Elements of Statistical Learning. Springer New York, 2009.

7. Heymans MW, van Buuren S, Knol DL, van Mechelen W, de Vet HC. Variable selection under multiple imputation using the bootstrap in a prognostic study. BMC Med. Res. Methodol. **2007**; 7:33.

8. van Buuren S. Flexible Imputation of Missing Data. Chapman and Hall/CRC, 2012.

9. Blanche P, Latouche A, Viallon V. Time-Dependent AUC with Right-Censored Data: A Survey. In: Lee M-LT, Gail M, Pfeiffer R, Satten G, Cai T, Gandy A, eds. Risk Assessment and Evaluation of Predictions. New York, NY: Springer New York, 2013: 239–251.

10. Harrell F. Regression Modeling Strategies With Applications to Linear Models, Logistic and Ordinal Regression, and Survival Analysis. Srpinger New York, 2015.

11. R Core Team. R: A Language and Environment for Statistical Computing. 2016;

12. Therneau TM. A Package for Survival Analysis in {S}. 2015;

13. Harrell Jr FE. rms: Regression Modeling Strategies. 2016;

14. Van Buuren S, Groothuis-Oudshoorn K. Multivariate Imputation by Chained Equations. J. Stat. Softw. **2011**; 45:1–67.

15. Friedman J, Hastie T, Tibshirani R. Regularization Paths for Generalized Linear Models via Coordinate Descent. J. Stat. Softw. **2010**; 33:1–22.

16. Wood SN. Fast stable restricted maximum likelihood and marginal likelihood estimation of semiparametric generalized linear models. J. R. Stat. Soc. **2011**; 73:3–36.

17. Mogensen UB, Ishwaran H, Gerds TA. Evaluating Random Forests for Survival Analysis Using Prediction Error Curves. J. Stat. Softw. **2012**; 50:1–23.

18. Blanche P, Dartigues J-F, Jacqmin-Gadda H. Estimating and Comparing time-dependent areas under receiver operating characteristic curves for censored event times with competing risks. Stat. Med. **2013**; 32:5381–5397.

19. McPherson, Winston Chang , Joe Cheng , JJ Allaire YX and J. shiny: Web Application Framework for R. **2017**;

Figure Legends

Supplementary Figure 1: Flow diagram.

Supplementary Figure 2: Calibration plots of candidate models by HIV status in internal and temporal validation. The circles and lines display the predicted 9 month mortality (from the prognostic model) versus the observed 9-month month mortality (based on Kaplan-Meier estimation) in ten patient groups defined by quantiles of the predicted event probabilities. For internal validation, blue crosses display bootstrap bias-corrected Kaplan-Meier estimates.

Supplementary Figure 3: Nomograms for the prediction of 9-month mortality based on the final prognostic models for the HIV-uninfected and HIV-infected TBM populations respectively including drug resistance information. To derive a prediction, locate the value of each predictor on the corresponding variable line, read the corresponding points assigned on the 0-100 scale and sum all of these points to a total point score. Then read the result on the Total Points scale and its corresponding prediction below. For HIV-infected nomogram, the cohort variable was chosen as the most recent trial [3] since this is most relevant for future prediction

Supplementary Table 1: Summary of included studies

| **Cohort** | **Dexamethasone trial** | **Fluoroquinolone trial** | **TBM HIV cohort** | **ART timing trial** | **Intensified treatment trial** |
| --- | --- | --- | --- | --- | --- |
| Main publication | Dexamethasone for the treatment of tuberculous meningitis in adolescents and adults [10]^1^ | A randomized pharmacokinetic and pharmacodynamics comparison of fluoroquinolones for tuberculous meningitis [11]^1^ | Clinical and microbiological features of HIV-associated tuberculous meningitis in Vietnamese adults [12]^1^ | Timing of initiation of antiretroviral therapy in human immunodeficiency virus (HIV)–associated tuberculous meningitis [13]^1^ | Intensified anti-tuberculosis chemotherapy for the treatment of Vietnamese adults with  tuberculous meningitis [3]^1^ |
| Recruitment period | 2001-2004 | 2003-2004 | 2004-2005 | 2005-2007 | 2011-2014 |
| Sample size  (HIV/no HIV) | 545 (98, 436)^2^ | 61 (3, 58) | 58 (58, 0) | 253 (253, 0) | 817 (349, 468) |
| Study design | RCT | RCT | Observational study | RCT | RCT |
| Randomized  Interventions | Two arms, double-blind  - Standard antituberculosis treatment  - Standard antituberculosis treatment plus adjunctive treatment with dexamethasone^3^ | Four arms, open label  - Standard antituberculosis treatment  - Standard antituberculosis treatment with oral dose of  + ciprofloxacin (750mg/12 hours),  + levofloxacin (500mg/12 hours), or  + gatifloxacin (400mg/24 hours)  for the first 60 days of treatment |  | Two arms, double-blind  - Immediate ART (initiated within 7 day of commencing tuberculosis treatment)  - Deferred ART (initiated after 2 months of tuberculosis treatment) | Two arms, , double-blind  - Standard antituberculosis treatment  - Standard antituberculosis treatment intensified with a higher dose of rifampicin (15mg/kg) and levofloxacin (20mg/kg) for the first 8 weeks of treatment |
| Inclusion criteria | - Age ≥ 14 years  - Clinical diagnosis of TBM | - Age ≥ 14 years  - Clinical diagnosis of TBM | - Age ≥15 years - Clinical diagnosis of TBM  - HIV infected | - Age ≥15 years  - Clinical diagnosis of TBM  - HIV infected | - Age ≥18 years  - Clinical diagnosis of TBM |
| Exclusion criteria | - Contraindication to corticosteroid use  - More than one dose of any corticosteroids prior to recruitment  - More than 30 days of antituberculosis chemotherapy immediately prior to recruitment  - Lack of informed consent | - Contraindication to fluoroquinolone use  - Pregnancy  - Lack of informed consent | - Proven alternative diagnosis | - Contraindication to ART or antituberculosis treatment  - Previous ART  - Antituberculosis treatment  8–30 days immediately prior to recruitment  - Pregnancy  - Proven alternative diagnosis  - Lack of informed consent | - Contraindication to fluoroquinolones of rifampicin use  - More than 7 days of antituberculosis treatment prior to recruitment  - Known or suspected MDR^4^-TBM  - Creatinine > 3x ULN  - Bilirubin > 2.5x ULN  - AST/ALT > 5x ULN  - Pregnancy  - Lack of informed consent |
| TBM diagnosis |  | Antituberculosis treatment for TBM initiated on decision of the treating clinician |  |  |  |
| - Definite TBM | AFB seen or *M.tb* cultured from CSF |  | AFB seen or *M.tb* cultured from CSF | AFB seen or *M.tb* cultured from CSF | AFB seen or *M.tb* cultured from CSF |
| - Probable TBM | One or more of the following:  - Suspected active TB on Xray  - AFB from other specimen  - Clinical evidence of other EPTB |  | One or more of the following:  - Suspected active TB on Xray  - AFB from other specimen  - Clinical evidence of other EPTB | One or more of the following:  - Suspected active TB on Xray  - AFB from other specimen  - Clinical evidence of other EPTB | One or more of the following:  - Suspected active TB on Xray  - AFB from other specimen  - Clinical evidence of other EPTB |
| - Possible TBM | At least four of the following:  - History of TB  - Duration of illness >5 days  - Altered consciousness  - Focal neurologic signs.  - Yellow CSF  - >50% lymphocytes in the CSF  - CSF/blood glucose ratio <0.5 |  |  | At least two of the following  - History of TB  - Duration of illness >5 days  - GCS <15  - Focal neurological signs  and at least two of the following  - Yellow CSF  - >50% lymphocytes in the CSF  - CSF/blood glucose ratio <0.5 | At least two of the following  - History of TB  - Duration of illness >5 days  - GCS <15  - Focal neurological signs  and at least two of the following  - Yellow CSF  - >50% lymphocytes in the CSF  - CSF/blood glucose ratio <0.5 |
| - No TBM | Another diagnosis confirmed by microbiologic or histopathological evaluation. |  |  | Another diagnosis confirmed by microbiologic or histopathological evaluation | Another diagnosis confirmed by microbiologic or histopathological evaluation |
| Confirmed TBM cases | 170(31.2%) | 39 (63.9%) | 54 (93.1%) | 158 (62.5%) | 407 (49.8%) |
| Follow up | 9 months | 9 months | 9 months | 12 months | 9 months |
| Primary outcome | 9-month mortality | Fluoroquinolone CSF penetration | Not pre-defined (observational study) | 9-month mortality | 9-month mortality |
| Main finding | Treatment with dexamethasone  was associated with a reduced risk of death (RR 0.69; 95%CI 0.52 to 0.92; P=0.01) | Levofloxacin had highest CSF penetration (median AUC_0-24_ 0.74; range, 0.58 to 1.03) | HIV associated TBM had high mortality (67.2%) and high drug resistance rates. Prognostic indicators were higher TBM grade, low serum sodium and decreased CSF lymphocyte percentage | Timing of ART did not improve survival. Early ART was associated with more grade 4 adverse events (102 vs 87 P=0.04) | Intensified anti-TB treatment did not improve 9-month survival (HR, 0.94; 95% CI, 0.73-1.22, P=0.66) |

Abbreviations: RCT= randomised controlled clinical trial, ART=antiretroviral therapy, MDR=multidrug resistance, ULN=upper limit of normal, AFB=acid-fast bacilli, *M.tb*= *Mycobacterium tuberculosis*, EPTB=extra pulmonary tuberculosis, GCS=Glasgow coma score, RR= relative risk, CI= confidence interval, HR= hazard ratio.

^1^ Reference numbers refer to the main manuscript.

^2^ 11 patients had an unknown HIV status

^3^ The detailed dexamethasone dosing schedule is given in the primary publication

^4^ MDR (multidrug resistance) is defined as resistance of *M. tb* to at least rifampin and isoniazid

Supplementary Table 2: Definition of candidate predictors

| **Variable name** | **Type** | **Definition** |
| --- | --- | --- |
| Cohort | Categorical | 5 levels:   - Dexamethasone trial - Fluoroquinolone trial - TBM HIV cohort - ART timing trial - Intensified treatment trial |
| **General patient information** | | |
| Age | Continuous | Patient’s age (years) |
| Sex | Binary | Gender: Male, Female |
| Weight | Continuous | Patient’s weight (kg) |
| **Treatment** |  |  |
| Dexamethasone | Binary | Receiving dexamethasone |
| On ART at enrolment | Binary | On ART at enrolment [in HIV-infected population only] |
| **Baseline clinical variables** | | |
| MRC Grade | Categorical | MRC grade:   - Grade I: Glasgow comma score (GCS) of 15, without any focal neurological sign - Grade II: GCS of 11 to 14, or a score of 15 with focal neurological signs - Grade III: GSC of 10 or lower |
| Illness duration at study entry | Continuous | TBM illness duration at study entry (day) |
| Previous TB treatment | Binary | History of previous TB treatment |
| Focal neurological signs | Binary | Focal neurological signs: presence of any neurological deficit including hemiplegia, paraplegia, or quadriplegia |
| Temperature | Continuous | Temperature (degrees Celsius) |
| Convulsion | Binary | Presence of seizures before enrollment |
| **Baseline laboratory variables** | | |
| Plasma sodium | Continuous | Concentration of sodium in blood (mmol/l) |
| CSF lymphocyte count | Continuous | Number of lymphocytes in CSF (cells/ mm^3^) |
| CSF protein | Continuous | Protein level in CSF (g/l) |
| CSF glucose | Continuous | Glucose level in CSF (mmol/l) |
| Ratio of blood glucose  and CSF glucose | Continuous | Ratio of glucose level in blood and glucose level in CSF |
| Peripheral blood CD4 count | Continuous | CD4 cell counts in blood (cell/mm^3^) [in HIV-infected population only] |
| **Baseline radiology variable** | | |
| Chest x-ray miliary TB | Binary | Chest x-ray result: miliary tuberculosis |

Supplementary Table 3: Univariable analyses for candidate predictors in the HIV-uninfected TBM population using Cox regression models

| **Characteristic** | **Alive at 9 months or lost to follow-up (N=732)** | | **Dead**  **(N=219)** | | **HR** | **95% CI** | **p-value** |
| --- | --- | --- | --- | --- | --- | --- | --- |
|  | **n** | **Summary statistic** | **n** | **Summary statistic** |  |  |  |
| Age [years]* | 732 | 38 (26,51) | 219 | 48 (35,66) | 1.28 | 1.20 - 1.38 | <0.001 |
| Sex: Female | 732 | 313(42.8%) | 219 | 93(42.5%) | 1.03 | 0.78 - 1.34 | 0.86 |
| Weight [kg]* | 732 | 47(42,53) | 219 | 45 (40,50) | 0.81 | 0.69 – 0.95 | 0.011 |
| MRC Grade § | 732 |  | 219 |  |  |  | <0.001 |
| - MRC Grade I |  | 288(39.3%) |  | 39(17.8%) | 1 |  |  |
| - MRC Grade II |  | 344(47.0%) |  | 104(47.5%) | 2.08 | 1.44 - 3.00 | <0.001 |
| - MRC Grade III |  | 100(13.7%) |  | 76(34.7%) | 4.72 | 3.21 - 6.95 | <0.001 |
| Illness duration at study entry [days]$ | 730 | 15 (10,23) | 218 | 17 (10,30) | 1.19 | 1.05 - 1.34 | 0.008 |
| Previous TB treatment: Yes | 708 | 51(7.2%) | 214 | 37(17.3%) | 2.25 | 1.58 - 3.21 | <0.001 |
| GCS | 732 | 15(13,15) | 219 | 13(8,15) | 0.82 | 0.79 - 0.85 | <0.001 |
| Focal neurological signs: Yes | 732 | 365(49.9%) | 219 | 152(69.4%) | 2.07 | 1.55 - 2.75 | <0.001 |
| Temperature [Celsius] | 731 | 37.8(37.2,38.5) | 219 | 37.6(37.0,38.5) | 0.95 | 0.82 - 1.11 | 0.56 |
| Convulsion: Yes | 731 | 19(2.6%) | 219 | 6(2.7%) | 1.18 | 0.53 - 2.66 | 0.68 |
| Dexamethasone: No | 732 | 142(19.4%) | 219 | 67(30.6%) | 1.73 | 1.30 - 2.31 | <0.001 |
| Plasma sodium [mmol/l]* | 648 | 130 (125,134) | 195 | 130 (124,134) | 1.00 | 0.83 - 1.21 | 0.99 |
| CSF lymphocyte count [cells/ mm^3^] $ | 711 | 114.00(40.65,218.34) | 208 | 47.21(15.92,144.00) | 0.82 | 0.77 - 0.87 | <0.001 |
| CSF protein [g/l] | 701 | 1.25(0.71,1.99) | 208 | 1.08(0.60,2.00) | 1.01 | 0.92 - 1.12 | 0.77 |
| CSF glucose [mmol/l] $ | 707 | 1.52(1.00,2.30) | 213 | 1.55(1.00,2.22) | 0.93 | 0.83 - 1.05 | 0.25 |
| Ratio of CSF glucose and blood glucose $ | 639 | 0.30(0.20,0.40) | 191 | 0.30(0.24,0.43) | 1.17 | 0.97 - 1.40 | 0.10 |
| Resistance | 732 |  | 219 |  |  |  | 0.003 |
| - No or other resistance |  | 169(23.1%) |  | 34(15.5%) | 1 |  |  |
| - Isoniazid resistant |  | 51(7.0%) |  | 12(5.5%) | 1.12 | 0.58 - 2.16 | 0.73 |
| - Rifampin resistant/MDR^1^ |  | 4(0.6%) |  | 6(2.7%) | 5.14 | 2.16 - 12.27 | <0.001 |
| - Unknown resistance |  | 508(69.4%) |  | 167(76.3%) | 1.59 | 1.10 - 2.30 | 0.014 |
| Chest x-ray miliary TB: Yes | 679 | 119(17.5%) | 211 | 46(21.8%) | 1.21 | 0.87 - 1.68 | 0.25 |

HR=hazard ratio, CI=confidence interval, MDR= multi-drug resistance. Summary statistics are frequency (%) for categorical variables and median (IQR) for continuous variables. Number of censored/lost to follow-up patients before 260 days was 44 (5%). Analysis based on subjects with non-missing data for the respective characteristic.

$: HR per 2-fold increase

*: HR per 10-unit increase

^1^: MDR (multidrug resistance) is defined as resistance of *M.tuberculosis* to at least rifampin and isoniazid

§ MRC Grade I (GCS 15; no focal neurological signs); MRC Grade II (GCS 11-14, or 15 with focal neurological signs); MRC Grade III (GCS≤10)).

Supplementary Table 4: Univariable analyses for candidate predictors in the HIV-infected TBM population using Cox regression models

| **Characteristic** | **Alive at 9 months or lost to follow-up (N=364)** | | **Dead (N=384)** | | **HR** | **95% CI** | **p-value** |
| --- | --- | --- | --- | --- | --- | --- | --- |
|  | **n** | **Summary statistic** | **n** | **Summary statistic** |  |  |  |
| Age [years]* | 364 | 31(27,36) | 383 | 30(26,35) | 0.85 | 0.73 - 0.98 | 0.029 |
| Sex: Female | 364 | 53(14.6%) | 384 | 55(14.3%) | 1.02 | 0.77 - 1.36 | 0.88 |
| Weight [kg]* | 363 | 48(44,52) | 381 | 45(40,50) | 0.66 | 0.58 - 0.77 | <0.001 |
| MRC Grade § | 364 |  | 383 |  |  |  |  |
| - MRC Grade I |  | 181(49.7%) |  | 80(20.9%) | 1 |  |  |
| - MRC Grade II |  | 142(39.0%) |  | 153(40.0%) | 2.05 | 1.56 - 2.68 | <0.001 |
| - MRC Grade III |  | 41(11.3%) |  | 150(39.1%) | 4.90 | 3.73 - 6.44 | <0.001 |
| Illness duration at study entry [days] $ | 364 | 15(9,30) | 373 | 15(9,30) | 1.04 | 0.95 - 1.14 | 0.42 |
| Previous TB treatment: Yes | 362 | 69(19.1%) | 374 | 79(21.1%) | 1.08 | 0.84 - 1.38 | 0.56 |
| GCS | 364 | 15(13,15) | 383 | 12(8,15) | 0.84 | 0.82 - 0.86 | <0.001 |
| Focal neurological signs: Yes | 362 | 130(35.9%) | 370 | 171(46.2%) | 1.39 | 1.13 - 1.70 | 0.002 |
| Temperature [Celsius] | 364 | 37.5(37.1,38.3) | 383 | 37.8(37.3,38.5) | 1.22 | 1.09 - 1.36 | <0.001 |
| Convulsion: Yes | 362 | 7(1.9%) | 373 | 18(4.8%) | 1.81 | 1.13 - 2.91 | 0.014 |
| Dexamethasone: No | 364 | 346(95.1%) | 384 | 348(90.6%) | 1.43 | 1.01 - 2.01 | 0.042 |
| Plasma sodium [mmol/l]* | 339 | 127 (124,132) | 355 | 126 (121,132) | 0.84 | 0.72 - 0.97 | 0.015 |
| CSF lymphocyte count [cells/ mm^3^] $ | 340 | 90.90(32.22,214.62) | 355 | 60.90(17.64,160.88) | 0.91 | 0.87 - 0.95 | <0.001 |
| CSF protein [g/l] | 351 | 1.30(0.70,1.90) | 364 | 1.25(0.69,1.82) | 0.94 | 0.86 - 1.03 | 0.20 |
| CSF glucose [mmol/l] | 351 | 1.80(1.20,2.31) | 365 | 1.62(1.12,2.34) | 1.06 | 0.99 - 1.12 | 0.09 |
| Ratio of CSF glucose and blood glucose $ | 306 | 0.31(0.22,0.41) | 334 | 0.30(0.21,0.43) | 1.06 | 0.94 - 1.19 | 0.32 |
| Resistance | 364 |  | 384 |  |  |  |  |
| - No or other resistance |  | 162(44.5%) |  | 114(29.7%) | 1 |  |  |
| - Isoniazid resistant |  | 42(11.5%) |  | 67(17.5%) | 1.59 | 1.18 - 2.16 | 0.002 |
| - Rifampin resistant/MDR^1^ |  | 3(0.8%) |  | 22(5.7%) | 4.11 | 2.59 - 6.50 | <0.001 |
| - Unknown resistance |  | 157(43.1%) |  | 181(47.1%) | 1.49 | 1.18 - 1.89 | <0.001 |
| Chest x-ray miliary TB: Yes | 333 | 65(19.5%) | 302 | 49(16.2%) | 0.84 | 0.62 - 1.14 | 0.26 |
| Peripheral blood CD4 count [cells/mm^3^]$ | 323 | 57(23,130) | 323 | 32(12,68) | 0.84 | 0.79 - 0.89 | <0.001 |
| On ART at enrolment | 364 | 80(22.0%) | 381 | 44(11.6%) | 0.54 | 0.39 - 0.73 | <0.001 |

HR=hazard ratio, CI=confidence interval. Summary statistics are frequency (%) for categorical variables and median (IQR) for continuous variables

Number of censored/lost to follow-up patients before 260 days was 54 (7%). Analysis based on subjects with non-missing data for the respective characteristic.

$: HR per 2-fold increase

*: HR per 10-unit increase

^1^: MDR (multidrug resistance) is defined as resistance of *M.tuberculosis* to at least rifampin and isoniazid

§ MRC Grade I (GCS 15; no focal neurological signs); MRC Grade II (GCS 11-14, or 15 with focal neurological signs); MRC Grade III (GCS≤10)).

Supplementary Table 5: Models based on variable selection which were not chosen as final models in HIV-uninfected and HIV-infected TBM populations

| **Variable** | **HIV-uninfected TBM** | | |  | **HIV-infected TBM** | | |
| --- | --- | --- | --- | --- | --- | --- | --- |
|  | **Backward selection model** | | |  | **Lasso selection model** | | |
|  | **HR** | **95% CI** | **p-value** |  | **HR** | **95% CI** | **p-value** |
| Age [years]* | 1.24 | 1.15 - 1.33 | <0.001 |  |  |  |  |
| Weight [kg]* |  |  |  |  | 0.93 | 0.90 - 0.96 | <0.001 |
| MRC Grade § |  |  |  |  |  |  |  |
| - MRC Grade I | 1 |  |  |  | 1 |  |  |
| - MRC Grade II | 1.93 | 1.33 - 2.79 | <0.001 |  | 1.89 | 1.44 - 2.49 | <0.001 |
| - MRC Grade III | 4.43 | 3.01 - 6.52 | <0.001 |  | 4.37 | 3.30 - 5.79 | <0001 |
| Dexamethasone: No | 1.72 | 1.29 - 2.29 | <0.001 |  |  |  |  |
| CSF lymphocyte count [cells/ mm^3^] ‡ | 0.85 | 0.80 - 0.91 | <0.001 |  |  |  |  |
| Peripheral blood CD4 count [cells/mm^3^] |  |  |  |  | 0.90 | 0.84 - 0.96 | 0.002 |
| On ART at enrolment [Yes] |  |  |  |  | 0.92 | 0.64 - 1.32 | 0.64 |
| Cohort |  |  |  |  |  |  |  |
| - Intensified treatment trial |  |  |  |  | 1 |  |  |
| - Dexamethasone trial |  |  |  |  | 1.59 | 1.13 - 2.23 | 0.008 |
| - TBM HIV cohort |  |  |  |  | 2.35 | 1.60 - 3.45 | <0.001 |
| - ART timing trial |  |  |  |  | 1.55 | 1.19 - 2.02 | 0.001 |

95% confidence intervals and p-value for final models do not take into account the uncertainty of model selection.

HR=hazard ratio, CI=confidence interval.

* HR per 10-unit increase.

‡ HR per 2-fold increase.

§ MRC Grade I (GCS 15; no focal neurological signs); MRC Grade II (GCS 11-14, or 15 with focal neurological signs); MRC Grade III (GCS≤10)).

Supplementary Table 6: Final Cox regression models for 9-month survival in each HIV population including drug resistance information. Estimates were pooled across multiply imputed datasets.

| **Variable** | **HIV-uninfected TBM population** | | | | | | |  | **HIV-infected TBM population** | | | | | | |
| --- | --- | --- | --- | --- | --- | --- | --- | --- | --- | --- | --- | --- | --- | --- | --- |
|  | **Full model** | | |  | **Final model**  **(model selected by the lasso)** | | |  | **Full model** | | |  | **Final model**  **(Model selected by stepwise backwards model selection)** | | |
|  | **HR** | **95% CI** | **p-value** |  | **HR** | **95% CI** | **p-value** |  | **HR** | **95% CI** | **p-value** |  | **HR** | **95% CI** | **p-value** |
| Age [per +10 years] | 1.23 | 1.14 - 1.33 | <0.001 |  | 1.23 | 1.14 - 1.32 | <0.001 |  | 0.96 | 0.82 - 1.12 | 0.64 |  |  |  |  |
| Sex: male | 1.04 | 0.77 - 1.41 | 0.78 |  |  |  |  |  | 0.98 | 0.72 - 1.35 | 0.92 |  |  |  |  |
| Weight [per +10 kgs] | 0.88 | 0.73 - 1.06 | 0.20 |  |  |  |  |  | 0.71 | 0.60 - 0.84 | <0.001 |  | 0.70 | 0.60 - 0.82 | <0.001 |
| MRC Grade § |  |  |  |  |  |  |  |  |  |  |  |  |  |  |  |
| - MRC Grade I | 1 |  |  |  | 1 |  |  |  | 1 |  |  |  | 1 |  |  |
| - MRC Grade II | 1.45 | 0.93 - 2.27 | 0.10 |  | 1.51 | 0.97 - 2.34 | 0.07 |  | 1.73 | 1.26 - 2.39 | <0.001 |  | 1.89 | 1.43 - 2.48 | <0.001 |
| - MRC Grade III | 3.32 | 2.03 - 5.43 | <0.001 |  | 3.43 | 2.14 - 5.48 | <0.001 |  | 3.95 | 2.82 - 5.53 | <0.001 |  | 4.22 | 3.18 - 5.60 | <0.001 |
| Illness duration at study entry [day] ‡ | 1.05 | 0.92 - 1.19 | 0.50 |  |  |  |  |  | 1 | 0.91 - 1.10 | 0.97 |  |  |  |  |
| Previous TB treatment: Yes | 1.38 | 0.93 - 2.05 | 0.11 |  | 1.49 | 1.02 - 2.16 | 0.037 |  | 1.16 | 0.88 - 1.52 | 0.28 |  |  |  |  |
| Focal neurological signs: Yes | 1.77 | 1.20 - 2.60 | 0.004 |  | 1.62 | 1.12 - 2.33 | 0.010 |  | 1.14 | 0.86 - 1.51 | 0.37 |  |  |  |  |
| Temperature[Celsius] | 0.95 | 0.78 - 1.16 | 0.64 |  |  |  |  |  | 1.09 | 0.96 - 1.24 | 0.20 |  |  |  |  |
| Convulsion: Yes | 0.89 | 0.37 - 2.13 | 0.79 |  |  |  |  |  | 1.01 | 0.61 - 1.66 | 0.98 |  |  |  |  |
| Dexamethasone: No | 1.71 | 1.19 - 2.5 | 0.004 |  | 2.0 | 1.45 - 2.70 | <0.001 |  | 1.04 | 0.63 - 1.72 | 0.88 |  |  |  |  |
| Plasma sodium [per +10 mmol/l] £ | 0.99 | 0.83 - 1.19 | 0.93 |  |  |  |  |  | - | - | - |  | - | - | - |
| - 135 vs. 125 | 0.99 | 0.83 - 1.19 |  |  |  |  |  |  | 1.08 | 0.89 - 1.30 |  |  | 1.08 | 0.90 - 1.30 |  |
| - 115 vs. 125 | 1.01 | 0.85 - 1.20 |  |  |  |  |  |  | 1.67 | 1.33 - 2.09 |  |  | 1.66 | 1.33 - 2.06 |  |
| CSF lymphocyte count [cells/ mm^3^] ‡ | 0.88 | 0.81 - 0.94 | <0.001 |  | 0.86 | 0.81 - 0.92 | <0.001 |  | 0.95 | 0.89 - 1.01 | 0.09 |  |  |  |  |
| CSF protein [g/l] ‡ | 0.97 | 0.85 - 1.10 | 0.60 |  |  |  |  |  | 0.96 | 0.86 - 1.07 | 0.47 |  |  |  |  |
| CSF glucose [mmol/l] | 0.99 | 0.83 - 1.19 | 0.95 |  |  |  |  |  | 1.05 | 0.95 - 1.15 | 0.32 |  |  |  |  |
| Ratio of CSF glucose and  blood glucose ‡ | 1.07 | 0.84 - 1.36 | 0.57 |  |  |  |  |  | 0.93 | 0.79 - 1.10 | 0.41 |  |  |  |  |
| Resistance ¥ |  |  |  |  |  |  |  |  |  |  |  |  |  |  |  |
| - No isoniazid or rifampin resistance | 1 |  |  |  | 1 |  |  |  | 1 |  |  |  | 1 |  |  |
| - Isoniazid monoresistance | 0.87 | 0.45 - 1.71 | 0.69 |  | 0.9 | 0.46 - 1.73 | 0.74 |  | 1.56 | 1.14 - 2.14 | 0.005 |  | 1.57 | 1.15 - 2.13 | 0.004 |
| - Rifampin monoresistance /MDR | 5.78 | 2.32 - 14.42 | <0.001 |  | 5.8 | 2.39 - 14.04 | <0.001 |  | 5.38 | 3.31 - 8.75 | <0.001 |  | 5.56 | 3.48 - 8.89 | <0.001 |
| - Unknown resistance | 1.37 | 0.93 - 2.03 | 0.12 |  | 1.47 | 1.01 - 2.14 | 0.046 |  | 1.66 | 1.28 - 2.16 | <0.001 |  | 1.74 | 1.37 - 2.22 | <0.001 |
| Chest x-ray miliary TB: Yes | 0.85 | 0.58 - 1.24 | 0.39 |  |  |  |  |  | 1.01 | 0.74 - 1.37 | 0.97 |  |  |  |  |
| Peripheral blood CD4 [cells/mm^3^] ‡ |  |  |  |  |  |  |  |  | 0.91 | 0.84 - 0.98 | 0.012 |  | 0.88 | 0.82 - 0.94 | <0.001 |
| On ART at enrolment [Yes] |  |  |  |  |  |  |  |  | 0.77 | 0.53 - 1.12 | 0.17 |  |  |  |  |
| Cohort |  |  |  |  |  |  |  |  |  |  |  |  |  |  |  |
| - Intensified trial | 1 |  |  |  |  |  |  |  | 1 |  |  |  | 1 |  |  |
| - Dexamethasone trial | 1.37 | 0.88 - 2.15 | 0.17 |  |  |  |  |  | 1.56 | 0.97 - 2.52 | 0.07 |  | 1.54 | 1.12 - 2.11 | 0.008 |
| - Fluoroquinolone trial | 1.02 | 0.46 - 2.28 | 0.95 |  |  |  |  |  | - | - | - |  | - | - | - |
| - TBM HIV cohort | - |  |  |  |  |  |  |  | 2.69 | 1.70 - 4.23 | <0.001 |  | 2.84 | 1.96 - 4.13 | <0.001 |
| - ART timing trial | - |  |  |  |  |  |  |  | 1.79 | 1.28 - 2.49 | <0.001 |  | 1.84 | 1.44 - 2.36 | <0.001 |

HR=hazard ratio, CI=confidence interval. 95% confidence intervals and p-value for final models do not take into account the uncertainty of model selection.

‡HR per 2-fold increase.

£ In HIV-infected subjects, the effect of sodium on mortality was significantly non-linear and modelled with a restricted cubic spline function with 2 degrees of freedom. To simplify interpretation of the corresponding regression coefficients, only HRs for two derived sodium contrasts from that model are given.

¥: Isoniazid monoresistance is defined as resistance to isoniazid but not to rifampin. Multidrug resistance (MDR) is defined as

resistance to at least isoniazid and rifampin. Unknown resistance is defined as drug-susceptibility test results not available. In all categories, resistance to other drugs may be present.

§ MRC Grade I (GCS 15; no focal neurological signs); MRC Grade II (GCS 11-14, or 15 with focal neurological signs); MRC Grade III (GCS≤10)).

Supplementary Table 7: Discrimination of candidate models for TBM mortality by HIV population including drug resistance information as measured by the area under the time-dependent ROC curve (AUC) at 9-months.

| **Model** | **Internal validation** | |  | **Temporal validation** |
| --- | --- | --- | --- | --- |
|  | **Apparent AUC (95% CI)*** | **Optimism Corrected AUC ‡** |  | **AUC (95% CI)** |
| **HIV-uninfected population** | | | | |
| Full model | 0.80 (0.76-0.83) | 0.77 |  | 0.78 (0.72-0.83) |
| Model selected by stepwise backwards model selection | 0.79 (0.75-0.82) | 0.77 |  | 0.76 (0.70-0.82) |
| Model selected by the lasso method (**) | 0.79 (0.76-0.83) | 0.77 |  | 0.80 (0.75-0.86) |
| MRC Grade § | 0.66 (0.62-0.70) | 0.66 |  | 0.70 (0.64-0.75) |
| GCS | 0.68 (0.64-0.72) | 0.68 |  | 0.68 (0.62-0.75) |
| **HIV-infected population** | | | | |
| Full model | 0.81 0.78 0.85 | 0.79 |  | 0.78 (0.73-0.84) |
| Model selected by stepwise backwards model selection (**) | 0.80 (0.77-0.84) | 0.79 |  | 0.78 (0.73-0.84) |
| Model selected by the lasso method | 0.81 (0.77-0.84) | 0.79 |  | 0.78 (0.73-0.84) |
| MRC Grade § | 0.70 (0.66-0.74) | 0.70 |  | 0.69 (0.63-0.75) |
| GCS | 0.71 (0.67-0.75) | 0.71 |  | 0.68 (0.62-0.74) |

CI: confidence interval

*Refers to performance estimated directly from the original 45 imputed datasets that was used to develop the prediction models.

‡ Adjusted performance corrected for over-optimism through internal bootstrap validation.

** Final simplified model.

§ MRC Grade I (GCS 15; no focal neurological signs); MRC Grade II (GCS 11-14, or 15 with focal neurological signs); MRC Grade III (GCS≤10)).

Supplementary figure 1


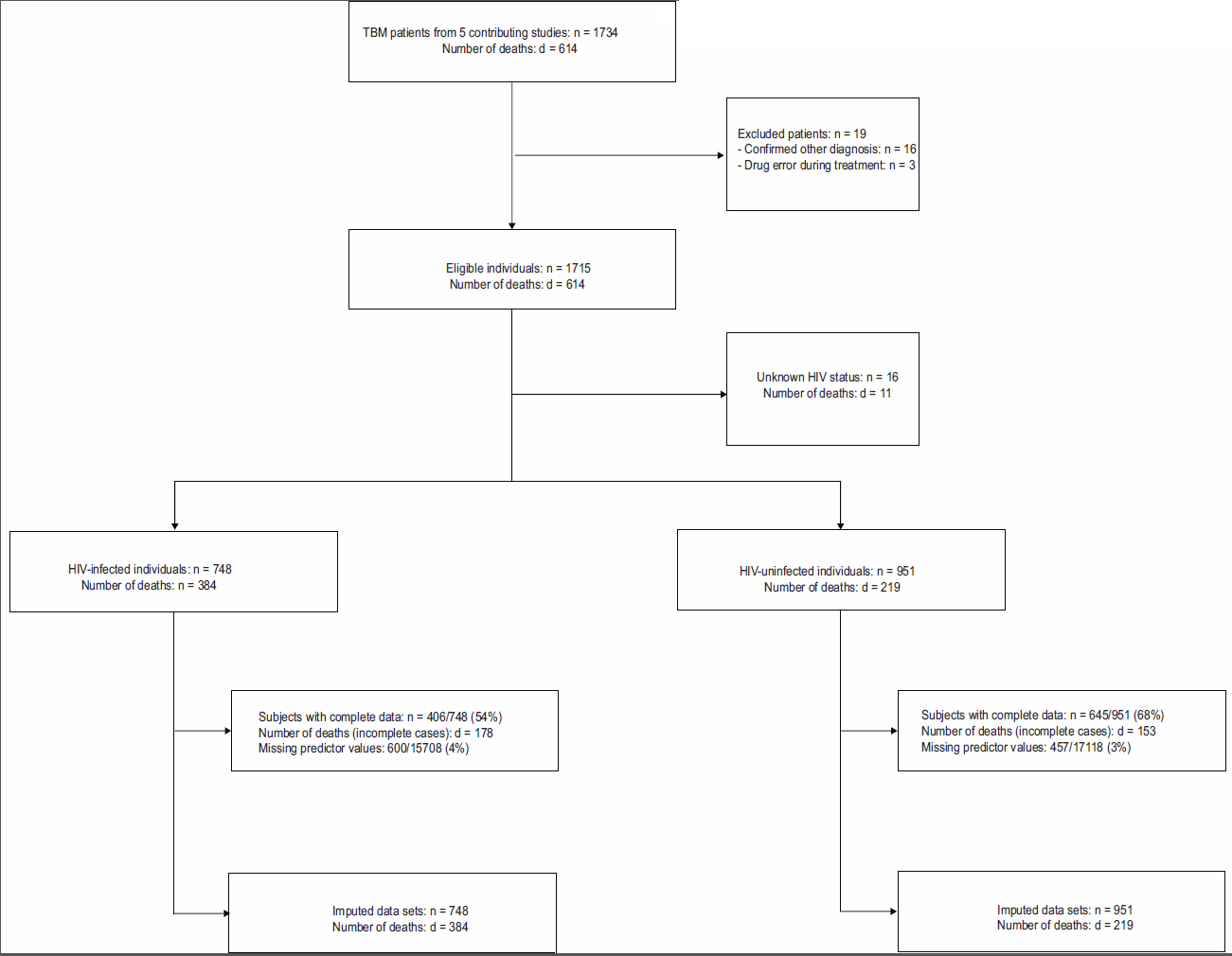


Supplementary figure 2


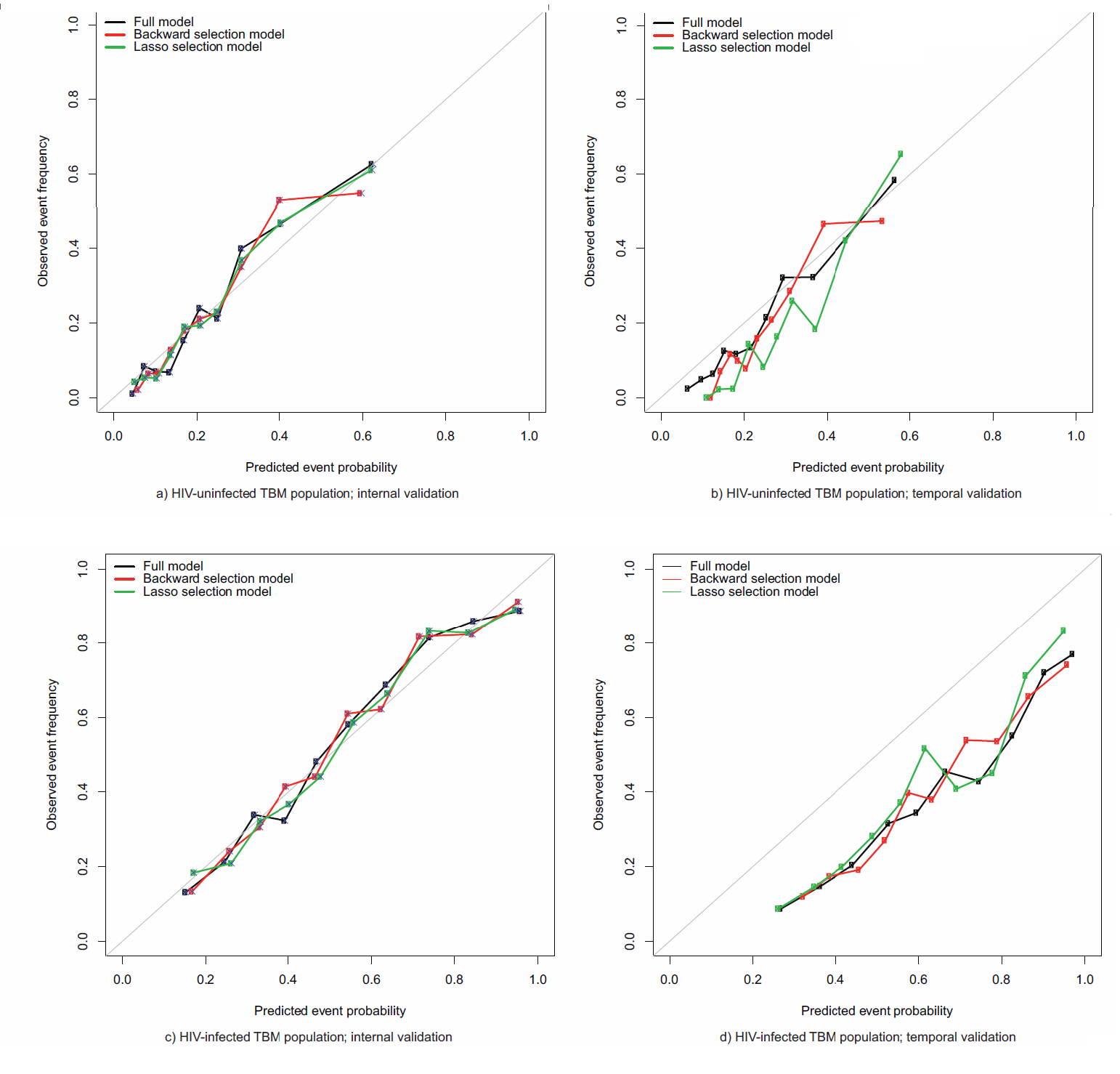


Supplementary figure 3
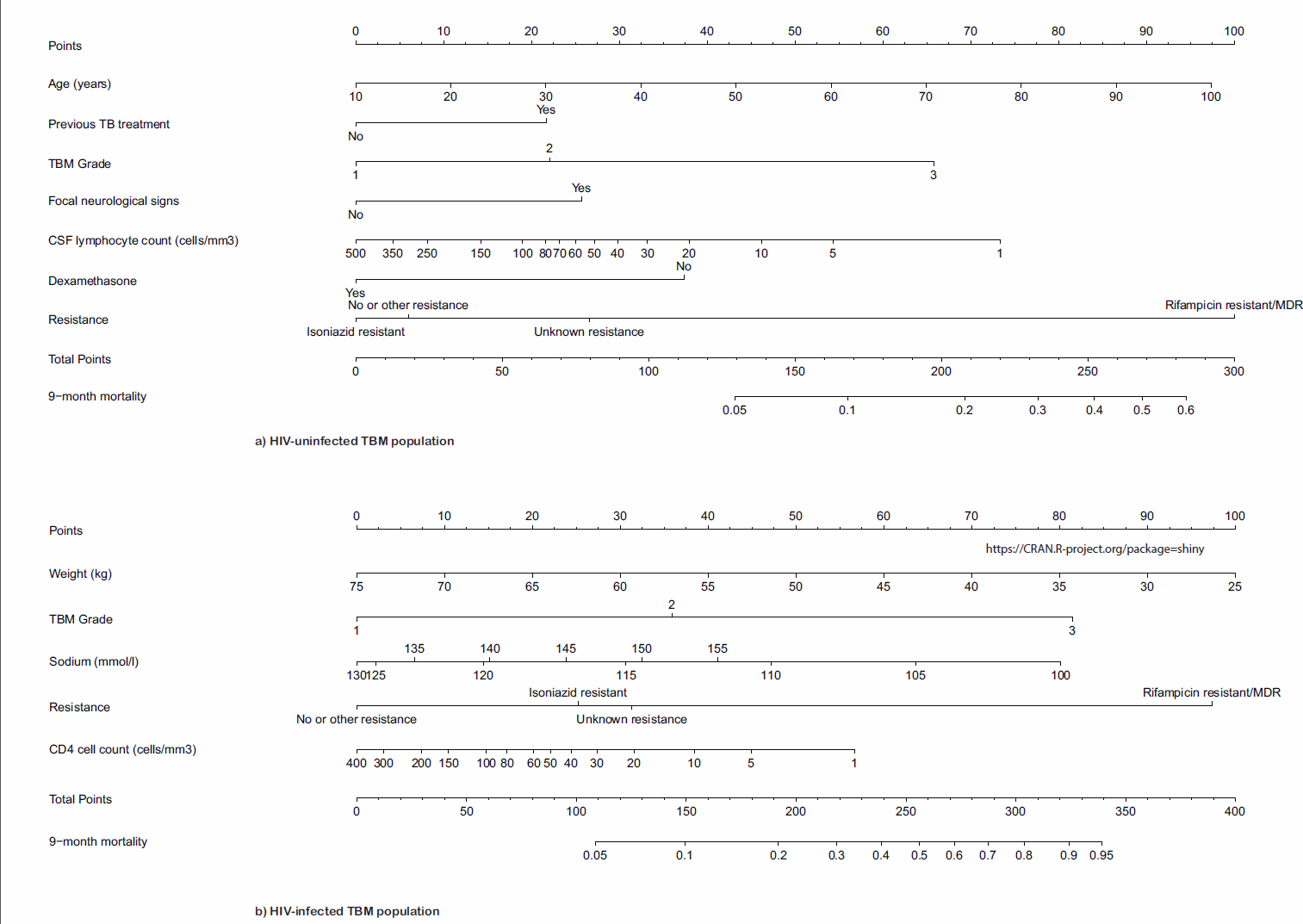

Supplement: Supplemental Data [file cix849_suppl_supplemental_data.docx]
